# Will the Interactions of Some Platinum (II)-Based Drugs with B-Vitamins Reduce Their Therapeutic Effect in Cancer Patients? Comparison of Chemotherapeutic Agents such as Cisplatin, Carboplatin and Oxaliplatin—A Review

**DOI:** 10.3390/ijms24021548

**Published:** 2023-01-12

**Authors:** Beata Szefler, Przemysław Czeleń

**Affiliations:** Department of Physical Chemistry, Faculty of Pharmacy, Collegium Medicum, Nicolaus Copernicus University, Kurpińskiego 5, 85-096 Bydgoszcz, Poland

**Keywords:** cisplatin, carboplatin, oxaliplatin, platinum-based drugs, cancer treatment, vitamin B

## Abstract

Pt (II) derivatives show anti-cancer activity by interacting with nucleobases of DNA, thus causing some spontaneous and non-spontaneous reactions. As a result, mono- and diaqua products are formed which further undergo complexation with guanine or adenine. Consequently, many processes are triggered, which lead to the death of the cancer cell. The theoretical and experimental studies confirm that such types of interactions can also occur with other chemical compounds. The vitamins from B group have a similar structure to the nucleobases of DNA and have aromatic rings with single-pair orbitals. Theoretical and experimental studies were performed to describe the interactions of B vitamins with Pt (II) derivatives such as cisplatin, oxaliplatin and carboplatin. The obtained results were compared with the values for guanine. Two levels of simulations were implemented at the theoretical level, namely, B3LYP/6-31G(d,p) with LANL2DZ bases set for platinum atoms and MN15/def2-TZVP. The polarizable continuum model (IEF–PCM preparation) and water as a solvent were used. UV-Vis spectroscopy was used to describe the drug–nucleobase and drug–B vitamin interactions. Values of the free energy (ΔG_r_) show spontaneous reactions with mono- and diaqua derivatives of cisplatin and oxaliplatin; however, interactions with diaqua derivatives are more preferable. The strength of these interactions was also compared. Carboplatin products have the weakest interaction with the studied structures. The presence of non-covalent interactions was demonstrated in the tested complexes. A good agreement between theory and experiment was also demonstrated.

## 1. Introduction

**Figure 1:**
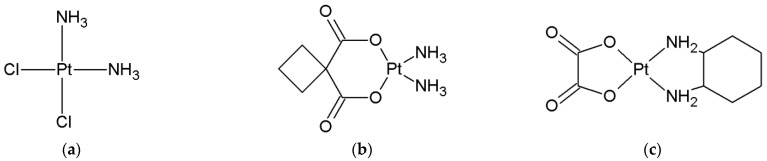
The structure of platinum (II)-based drugs (**a**) cisplatin, (**b**) carboplatin and (**c**) oxaliplatin.

Cisplatin, oxaliplatin and carboplatin belong to the same group of anticancer agents, Pt (II) drugs, which contain a heavy metal, i.e., platinum, and have anticancer activity (Figure 1) [1,2,3,4,5,6]. Cisplatin (cis-diamminedichloroplatinum) and other drugs under consideration are metallic (platinum) coordination compounds with a square planar geometry (Figure 1). Carboplatin (Figure 1b) is a 1,1-cyclobutyldicarboxylat, while oxaliplatin (Figure 1c) is a complex with 1,2-diaminocyclohexane and an oxalate group (DACH) [1,2,3,4,5,6].

Cisplatin is a chemotherapeutic agent that has activity against a wide range of cancers [7,8], such as lung, ovarian, testicular, bladder, head and neck cancers. It is a drug that has shown high anticancer efficacy against various types of cancer, including carcinomas, lymphomas, germ cell tumors and sarcomas. Carboplatin found its use especially in the treatment of cancer of the ovary, testis, head, neck and small cell lung cancer [9]. Compared to cisplatin, carboplatin is less toxic but therefore less therapeutic and must be used at several times higher doses. It can be used as a monotherapeutic or in combination therapy with another agent [10,11,12,13]. Oxaliplatin shows anticancer equivalence to cisplatin in the treatment of esophageal and gastric cancer [14]. It is an alkylating and a cytostatic drug used in chemotherapy of malignant tumors mainly of colorectal cancer [14,15,16,17,18,19,20,21,22,23]. Oxaliplatin is used against a broad spectrum of tumors, including some cisplatin- and carboplatin-resistant cell lines [24,25].

Cisplatin, oxaliplatin and carboplatin have a similar mechanism of action [26,27,28,29,30,31,32,33,34,35] and work by interacting with the cell’s genetic material, i.e., DNA nucleic acid (deoxyribonucleic acid).

**Figure 2:**
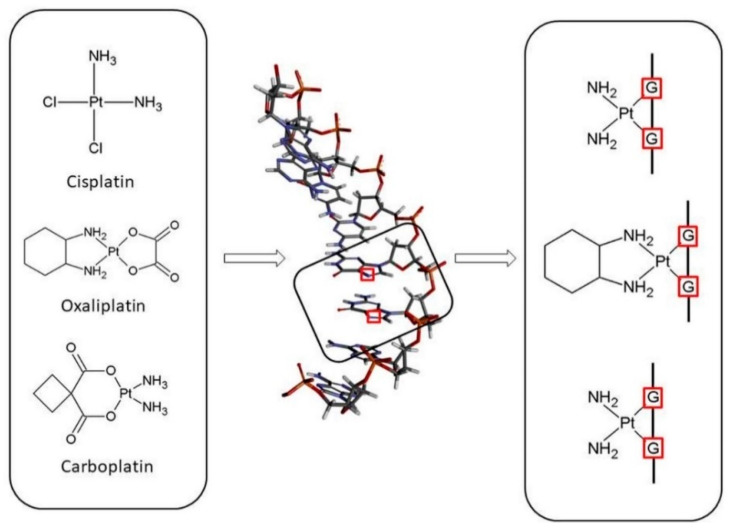
Scheme of interactions of platinum (II)-based drugs (cisplatin, carboplatin and oxaliplatin) with nucleobases from DNA.

These three Pt (II) drugs react with DNA by having the ability to crosslink with the canonical purine bases, primarily with guanine or adenine but in the case of oxaliplatin also with cytosine, and form crosslinks both within the molecule and between the DNA molecules (Figure 2) [22,31,36]. The adducts formed by these compounds can be mono adducts or intra- and interchain di adducts. The formation of incorrect bonds causes structural changes in the DNA strand, causing its breakage and thus damage to the DNA strand, disrupts DNA synthesis, prevents cell division, and then induces apoptosis in cancer cells [9,31,37,38,39,40].

**Figure 3:**
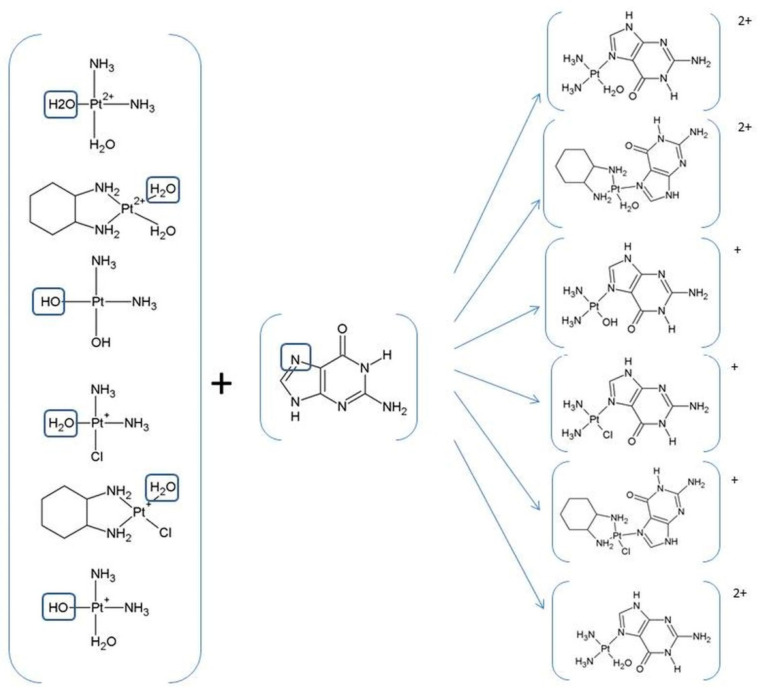
Scheme of complexes of guanine with mono-(di-)aqua and mono-(di-)hydroxy derivatives of three platinum (II)-based drugs.

The three drugs pass through the cell membrane via passive diffusion or active transport via copper transport CTR1 [41,42,43,44]. After entering the cell, the drug in the cell nucleus interacts with the nitrogen atom N_7_ of the nucleobase, i.e., guanine or adenine, to form a monoadduct and then a di adduct (Figure 3) [45,46].

**Figure 4:**
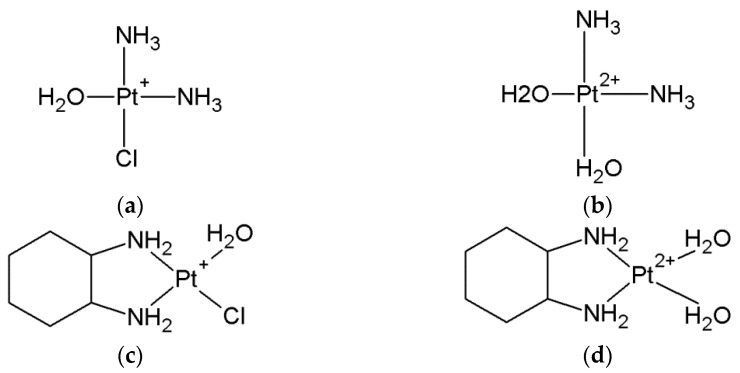

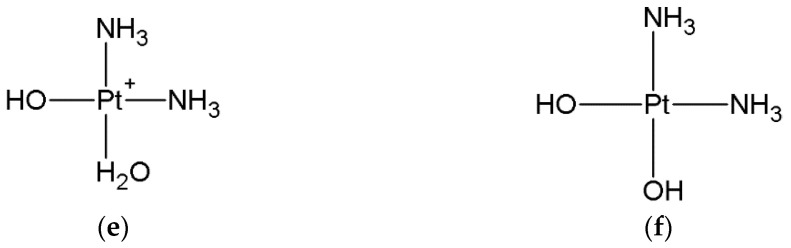
The products of hydrolysis of platinum (II)-based drugs as monoaqua (**a**) cis~Pt-Chloroaqua, (**c**) Pt-DACH-Chloroaqua, diaqua (**b**) cis~Pt-diaqua, (**d**) Pt-DACH-diaqua, monohydroxy (**e**) Pt(NH_3_)_2_H_2_O(OH)^+^ and dihydroxy derivatives (**f**) Pt(NH_3_)_2_(OH)_2_.

Pt (II) drugs are administered to the patient in an inactive therapeutic form and in order to interact with the nucleobases in DNA. They must undergo a series of reactions (Figure 2, Figure 3 and Figure 4).

Cisplatin and oxaliplatin have a similar mechanism of action where for the activation of the relatively inert platinum (II) complexes, the hydrolysis of non-active drugs to diaqua complexes via the monoaqua form is necessary [47,48,49,50,51,52] (Figure 2, Figure 3 and Figure 4).

Inside the tumor cell, the molecule of cisplatin (oxaliplatin) undergoes hydrolysis wherein the chlorine ligand is replaced by a water molecule and forms monoaqua derivatives (cis~Pt-monoaqua) (Figure 3 and Figure 4). This process occurs because of the low concentration of chloride ions (~3–20 mM) inside the cell. In the next step, monoaqua derivatives (cis~Pt-monoaqua, Pt-DACH-monoaqua) spontaneously transform to diaqua forms (cis~Pt-diaqua, Pt-DACH-diaqua) (Figure 3). The second step of hydrolysis occurs much more slowly (K = 2.75∙10^−5^s^−1^) compared to the first (K = 5.18∙10^−5^s^−1^) [45,53] in the case of cisplatin [47].

For the decomposition of carboplatin in water, two steps of the mechanism are needed. In the first one, the ring of 1,1-cyclobutanedicarboxylate must be opened where the malonato ligand is lost. The bond breakage between the platinum and the oxygen atoms in the carboplatin leads to the formation of a molecule with a total charge of one (Figure 3 and Figure 4) [54]. Because of the presence of a water molecule, the monohydroxy derivatives of carboplatin [(NH_3_)_2_Pt(OH)H_2_O]^+^ undergo further hydrolysis to dihydroxy derivatives (NH_3_)_2_Pt(OH)_2_, which via dihydroxylation form [Pt(NH_3_)_2_(OH)]^+^ (Figure 3 and Figure 4).

Monoaqua and diaqua derivatives of Pt (II) drugs such as cisplatin, carboplatin and oxaliplatin can easily generate the formation of adducts of platinum by interacting with nucleophilic molecules within the cell, such as DNA, RNA, and proteins [17,18,55].

### Motivation of Study

**Figure 5:**
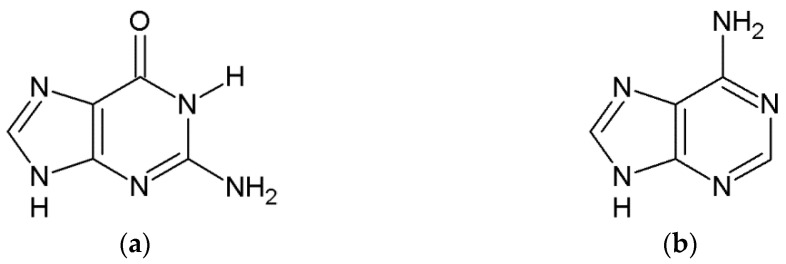

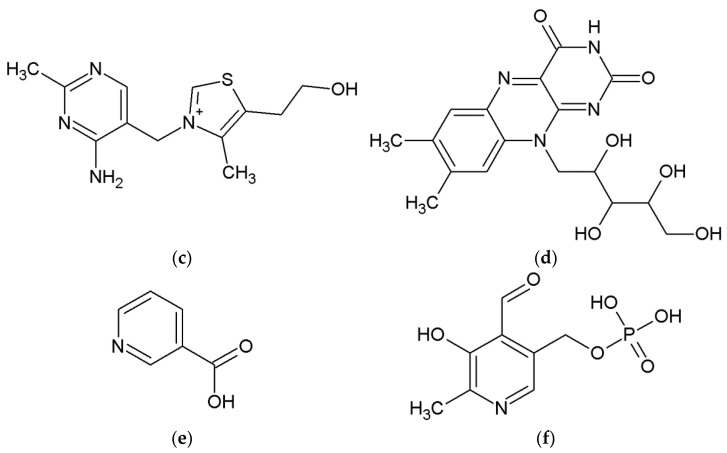
The structure of compounds used in reactions with platinum (II)-based drugs (**a**) guanine, (**b**) adenine (**c**) thiamine, (**d**) riboflavin, (**e**) niacin and (**f**) pyridoxal phosphate.

**Figure 6:**
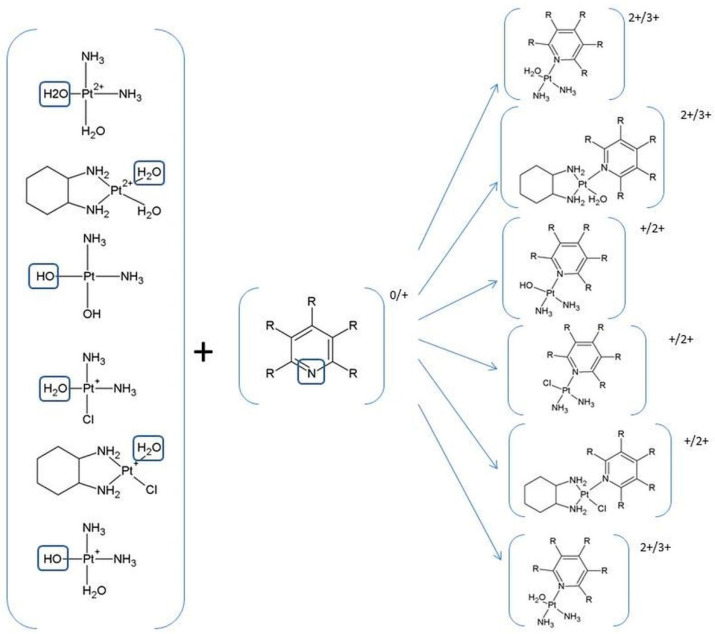
Scheme of complexes of B vitamins with mono-(di-)aqua and mono-(di-)hydroxy derivatives of three platinum (II)-based drugs.

During the study, the reactivity of hydrolysis products such as monoaqua and diaqua derivatives of three platinum (II)-based drugs, cisplatin, carboplatin and oxaliplatin, was analyzed [47,48,49,50,51,52]. Such products as [(cis~[Pt(NH_3_)_2_Cl(H_2_O)]^+^) and (cis~[Pt(NH_3_)_2_(H_2_O)_2_]^2+^)] in the case of cisplatin, [Pt(H_2_O)ClDACH]^+^ and [Pt(H_2_O)_2_DACH]^2+^ in the case of oxaliplatin and Pt(NH_3_)_2_H_2_O(OH)^+^ plus Pt(NH_3_)_2_(OH)_2_ in the case of carboplatin can interact not only with nucleobases (Figure 3 and Figure 4), which is the basis of their therapeutic action, but also with all compounds which have aromatic rings in their structures with lone-pair orbitals analogous to N_7_ in purine (Figure 5 and Figure 6). The group of such compounds includes vitamins from B group (Figure 5), namely, vitamins B1, B2, B3 and B6, with their alternative names thiamine, riboflavin, niacin and pyridoxal phosphate [1,56,57,58,59,60,61,62,63], respectively.

B vitamins not only occur naturally in some food products, but mainly their production takes place through synthesis. These B vitamins are contained in many elements of the daily diet, first of all, in vegetables such as carrot, beetroot and tomato, and in large significant doses in their juices and purees [1,56,57,58,59,60,61,62,63]. B vitamins are supplemented in many disorders of the human body. Vitamin B1 (thiamine) is a heterocyclic chemical compound composed of thiazole and pyrimidine rings connected by a methylene bridge. Thiamine participates in glucose metabolism in the human body [1,56,57,58,59,60,61,62,63]. The substance is used in states of vitamin B1 deficiency. It is used in the treatment of beriberi disease, Wernicke’s encephalopathy, neuropathies, in the course of diabetes or alcoholism and pain syndromes in rheumatology and neurology. Prophylactically, thiamine is used in states of increased demand for this vitamin, i.e., hyperthyroidism, improper diet, chronic infections and fever, alcoholism, excessive physical effort, persistent diarrhea, general weakness and mental and physical exhaustion. Vitamin B2 (riboflavin) is an organic chemical compound, a combination of ribitol and flavin [1,56,57,58,59,60,61,62,63]. In the human body it acts as a vitamin, the deficiency of which can cause disorders in the functioning of the nervous system and inflammation of the mucous membranes. Vitamin B2 (Riboflavin) occurs in the form of riboflavin-5′-phosphate, which is gradually broken down in the body to riboflavin after ingestion. Vitamin B2-riboflavin-5′-phosphate is sought after by people oriented to conditions of the skin, eyesight and mucous membranes, for support for proper energy metabolism, support for iron absorption, support for the nervous system and mental condition and support in reducing tiredness and fatigue. Niacin, also known as nicotinic acid, is an organic compound and a form of vitamin B3, an essential human nutrient [1,56,57,58,59,60,61,62,63]. It can be produced by plants and animals from the amino acid tryptophan. It contributes to the maintenance of proper energy metabolism, helps in the proper functioning of the nervous system and maintains normal psychological functions. The role of niacin in the human body is wide. Namely, it regulates the burning of fats, proteins and carbohydrates, is necessary for the proper functioning of the brain and peripheral nervous system, participates in the synthesis of neurotransmitters, has antidepressant properties, lowers the level of cholesterol and triglycerides in the blood, dilates blood vessels, lowers blood pressure, regulates the work of the digestive tract (its motor and secretory functions), regulates the function of the liver and pancreas, participates in the synthesis of sex hormones, cortisol, thyroxine and insulin, participates in the formation of erythrocytes, participates in the detoxification processes of the body, improves the appearance of the skin, hair and nails, prevents pellagra and accelerates wound healing. Pyridoxal phosphate (vitamin B6), acts as a coenzyme or prosthetic group and is necessary for the operation of many enzymes such as enzymes involved in the biosynthesis of amino acids and amino acid-derived metabolites, but also enzymes found in the biosynthetic pathways of amino sugars and in the synthesis or catabolism of neurotransmitters [1,56,57,58,59,60,61,62,63]. Vitamin B6 can also inhibit DNA polymerases and several steroid receptors. Inadequate levels of pyridoxal phosphate in the brain can cause neurological dysfunction, particularly epilepsy.

Thus, B vitamins can be a competitive element for nucleobases of DNA, with which these drugs should ultimately bind. The schemes of interactions of the monoaqua and diaqua derivatives of three platinum (II)-based drugs, cisplatin, carboplatin and oxaliplatin, with nucleobases (Figure 2 and Figure 3) and vitamins from B group are given below (Figure 6).

## 2. Results

**Figure 7:**
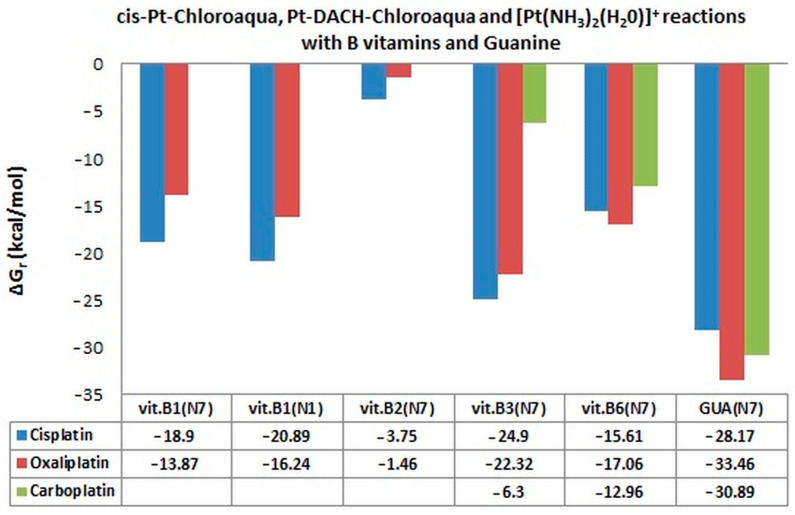
Gibbs free energy (ΔG_r_) values in kcal/mol of complexes of monoaqua derivatives of cisplatin, oxaliplatin and monohydroxy derivatives of carboplatin with B vitamins and guanine. Only negative values of ΔG_r_ (kcal/mol) are included in the figure at B3LYP/6-31G(d,p)/LANL2DZ level of theory.

**Figure 8:**
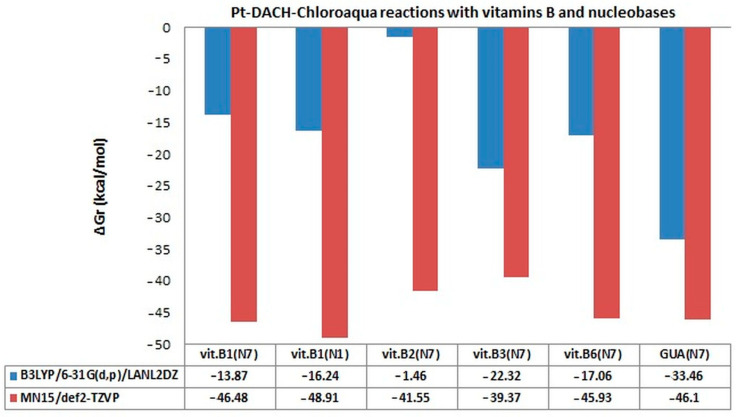
Gibbs free energy (ΔG_r_) values in kcal/mol of complexes of monoaqua derivatives of oxaliplatin (Pt-DACH-Chloroaqua) with B vitamins and guanine at B3LYP/6-31G(d,p)/LANL2DZ and MN15/def2-TZVP level of theory [52].

**Figure 9:**
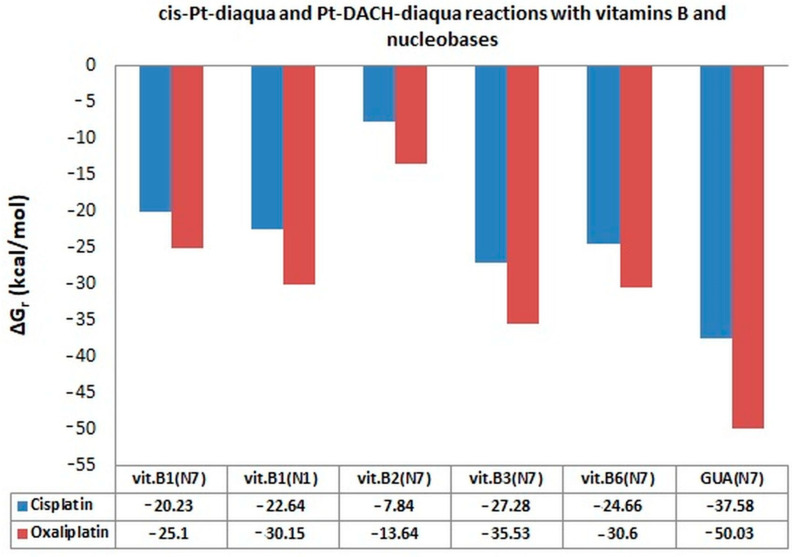
Comparison of Gibbs free energy (ΔG_r_) values in kcal/mol of complexes of diaqua derivatives of cisplatin and oxaliplatin (cis-Pt-diaqua and Pt-DACH-diaqua) at B3LYP/6-31G(d,p)/LANL2DZ level of theory [49,52].

**Figure 10:**
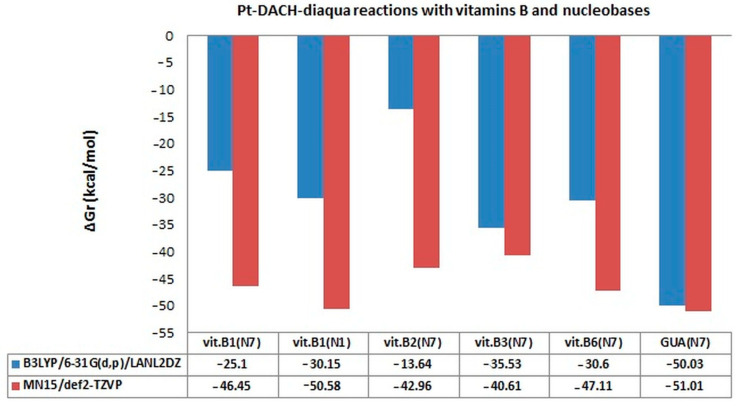
Gibbs free energy (ΔG_r_) values in kcal/mol of complexes of diaqua derivatives of oxaliplatin (Pt-DACH-diaqua) with B vitamins and guanine at B3LYP/6-31G(d,p)/LANL2DZ and MN15/def2-TZVP level of theory [52].

In their structure, the B vitamins have a purine and/or pyrimidine rings and thus they have aromatic ring/rings with lone-pair orbitals analogous to N_7_ in purine. Thiamine (vitamin B1) has the second addition center for interaction such as the nitrogen atom N_1_. The affinities of all vitamins to cis-platinum were analyzed for the first time [49,50,51,52]. The estimated values of Gibbs free energy (ΔG_r_) of reaction for mono- and diaqua platinum complexes of the studied vitamins were compared with the results for guanine in this review (Figure 7, Figure 8, Figure 9 and Figure 10, Table 1, Table 2, Table 3 and Table 4).

The energetic characteristics of the cis-Pt-Chloroaqua and Pt-DACH-Chloroaqua reactions with B vitamins and nucleobases obtained at the B3LYP/6-31G(d,p)/LANL2DZ level of theory are similar (Table 1, Figure 7) in water solution. The order of affinities of the studied vitamins to the drugs cisplatin and oxaliplatin are the same, from B3 vitamin via B1(N1 → N7) and B6 to B2 vitamin (Table 1, Figure 7). In the case of oxaliplatin, the order is slightly changed by vitamin B6. Among all B vitamins, B3 (niacin) most easily forms complexes with the hydrolysis products of the studied drugs, where ΔG_r_ values equal to −24.90 kcal/mol and −22.32 kcal/mol for cis~[Pt(NH_3_)_2_Cl]^+^ and for [PtClDACH]^+^, respectively (Table 1, Figure 7). Vitamin B2 (riboflavin) has the worst ability to create complexes with cis-Pt-Chloroaqua and Pt-DACH-Chloroaqua with values of Gibbs free energy equal to −3.75 kcal/mol and −1.46 kcal/mol (Table 1, Figure 7), respectively. Guanine has the highest affinity for both studied drugs, which was expected (Table 1, Figure 7); ΔG_r_ equals −28.17 kcal/mol in the case of cisplatin and −33.46 kcal/mol in the case of oxaliplatin. Quite good affinity is shown by guanine also with the product of hydrolysis of carboplatin with a value of Gibbs free energy equal to −30.89 kcal/mol.

Relative affinity to carboplatin is shown by B6 vitamin and then B3 vitamin with ΔG_r_ values equal to −12.96 kcal/mol and −6.3 kcal/mol, respectively. The other tested vitamins from B group have no affinity for this drug and their Gibbs free energy values are positive (Table 1, Figure 7).

As seen in Figure 7, most of the B vitamins interact less with chloroaqua (monoaqua) products of oxaliplatin compared to cisplatin. Guanine forms bonds with chloroaqua products of cisplatin, oxaliplatin and carboplatin more easily compared to B vitamins. Products of hydrolysis of carboplatin have the weakest interaction with the tested vitamins.

The change of the calculation level to a higher level, such as MN15/def2-TZVP, in the case of monoaqua products of oxaliplatin causes the change of the order of affinity for the studied vitamins as follows: vit. B1_(N1)_ → vit. B1_(N7)_ → vit. B6→ vit. B2 → vit. B3 (Table 2, Figure 8). The values of Gibbs free energy of B1_(N1)_ vitamin and guanine are similar, −46.48 kcal/mol and −46.10 kcal/mol, respectively.

Increasing the calculation level of calculate increases the values of Gibbs free energy (Table 2, Figure 8).

Unexpectedly, guanine does not have the greatest affinity for the drug (Table 2, Figure 8) and takes the value of Gibbs free energy equal to −46.10 kcal/mol. Vitamin B1 shows the greatest affinity to the drug, with particular participation in the reaction coming from the N_1_ nitrogen atom in the aromatic ring of the structure. Figure 8 shows that increasing the level of calculation from B3LYP/6-31G(d,p)/LANL2DZ to MN15/def2-TZVP significantly increases the values of ΔG_r_ of all B vitamins and guanine even two or three times (Table 2, Figure 8). The most spectacular affinity increase was observed for B2 vitamin, approximately a thirty-fold increase in the value of Gibbs Free Energy from −1.46 kcal/mol to −41.55 kcal/mol and at about 96.5%. Even so, in the case of other B vitamins, equally large increases were observed, i.e., over 40% for B3 vitamin, 63% for vitamin B6, 67% for B1_(N1)_ vitamin and 70% for B1_(N7)_ vitamin. This level of calculation clearly changes the affinity of B vitamins to the drug. It can be seen that vitamin B2 has the highest affinity for Oxaliplatin (−41.55 kcal/mol) compared to the B3LYP level of calculation (−1.46 kcal/mol), and its value does not significantly differ from the Gibbs Free Energy value for other vitamins from B group and Guanine.

Vitamin B3 (Niacin) easily forms complexes with cis-Pt-diaqua and Pt-DACH-diaqua, ([cis~Pt(NH_3_)_2_(H_2_O)_2_]^2+^ and [Pt(H2O)_2_DACH]^2+^), where delta Gibbs Free Energies are equal −27.28 kcal/mol and −35.53 kcal/mol in water solution, respectively. The diaqua forms, compared to the chloro- forms (Table 3, Figure 9) have a similar affinity for B vitamins and Guanine (GUA → vit. B3 → vit. B6 →vit. B1_(N1)_ → vit. B1_(N7)_ → vit. B2) at the B3LYP/6-31G(d,p)/LANL2DZ level of theory in water solution. The diaqua forms of Cisplatin and Oxaliplatin have the greatest affinity for Guanine (−37.58; −50.03) compared to B vitamins which show a stronger drug effect. Oxaliplatin hydrolysis products compared to Cisplatin show a higher affinity for both kinds of studied structures, namely nucleobases and B vitamins (Table 3, Figure 9). Vitamin B2 forms the weakest bonds with the diaqua products of Cisplatin and Oxaliplatin.

Diaqua forms of oxaliplatin show a higher affinity to B vitamins and guanine compared to cisplatin (Table 3, Figure 9). The highest increase in affinity was observed for guanine, more than 12 times, and next for vitamin B3, more than eight times. However, the order of affinity of both drugs to the tested vitamins and bases is the same.

As for Pt-Chloroaqua derivatives of the drugs, also in this case, increasing the calculation level causes the increase in the values of Gibbs free energy (Table 4, Figure 9 and Figure 10). In the case of diaqua forms of oxaliplatin, the order of affinity for studied vitamins and nucleobases is the same, GUA → vit. B1_(N1)_ → vit. B6→vit. B1_(N7)_ → vit. B2 → vit. B3, as for Pt-Chloroaqua derivatives. However, here the differences in values of ΔG_r_ between vitamin B1_(N7)_ and guanine are larger by about 0.43 kcal/mol.

The transition from a lower level of calculation to a higher one, that is from B3LYP/6-31G(d,p)/LANL2DZ to MN15/def2-TZVP, causes the greatest increase of affinities for B2 vitamin and B1 vitamin (Table 3, Figure 10) in the case of diaqua derivatives. For vitamin B3 there was a three-fold increase (68%) and for vitamins B1_(N7)_ and B1_(N1)_ an almost two-fold increase (46%; 40%).

The possibility of drug interaction with macromolecules may be indicated by such a physicochemical parameter as a change in the value of the molar volume of the formed vitamin B (nucleobases)–drug complexes, which will be directly related to conformational changes of the substrates of the complexation reaction. Such studies at the calculation level MN15/def2-TZVP were carried out for the effects of oxaliplatin and vitamins from B group and such nucleobases as guanine. The values of molar volume (cm^3^/mol) of studied complexes PtClDACH-Gua and PtH_2_ODACH-Gua are 198.370 cm^3^/mol and 210.671 cm^3^/mol, respectively.

**Figure 11:**
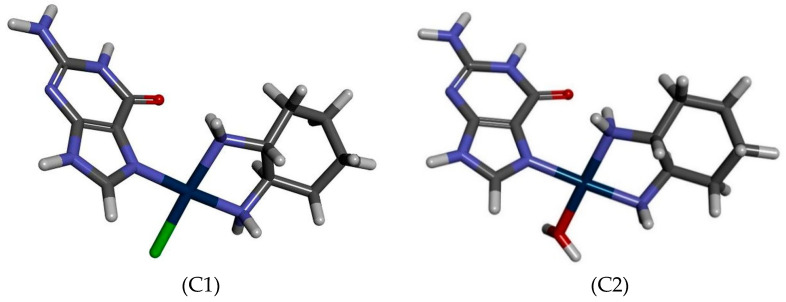
Graphic representation of PtClDACH-Gua (C1) and PtH_2_ODACH-Gua (C2) complexes [52].

In order to carry out the topological and electronic studies of the structural changes after the replacement of a chlorine atom with a water molecule, AIM studies were carried out (Figure 11, Table 5). The BCPs were found for non-covalent and covalent interactions (Figure 11).

An analysis of the interactions of the Pt atom with its immediate surroundings was carried out. In the PtClDACH-Gua complex BCP intramolecular interactions between Pt and Cl atoms from oxaliplatin and between O atom from guanine and H atom from the NH_2_ group of oxaliplatin moiety were found. In the case of PtH_2_ODACH-Gua, the interactions between the Pt atom of the drug with the N atom and an O atom from the water molecule and two N atoms from guanine were found. There is a BCP between an oxygen atom from guanine and a hydrogen atom from the NH_2_ group of the oxaliplatin moiety. This part of the study showed the network of interactions stabilizing the complexes. To describe the strength of the interactions, the potential electronic energy density was calculated. The BCP values were found between the Pt atom of the drug and the studied reagents. The electron density and its Laplacian values were changed with the appearance of a Cl atom or a H_2_O molecule in the vicinity of the Pt atom of oxaliplatin.

**Figure 12:**
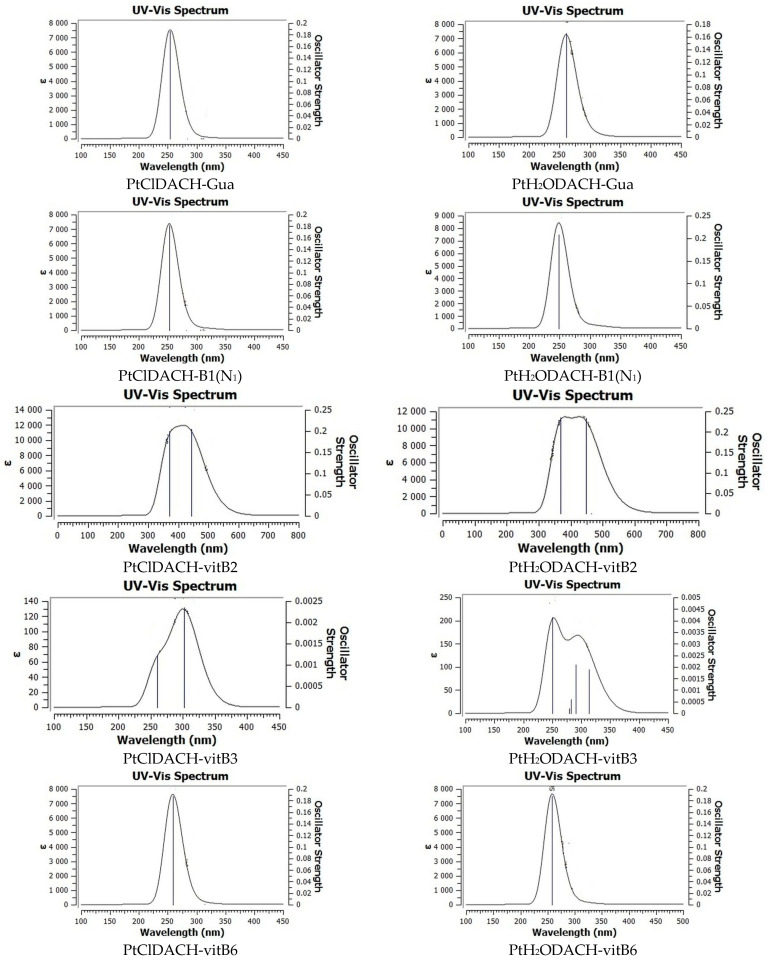
UV-Vis spectra calculated at MN15/def2-TZVP level of theory with PCM model and water as a solvent for selected oxaliplatin complexes with guanine and B vitamins [52].

To study the spectroscopic properties of drug–vitamin B or drug–guanine complexes, the TD-DFT (time-dependent density functional theory) was performed (Figure 12). The results were compared with experimental data (Section 3).

In Figure 12, the excitation energies are given for singlet-singlet transitions (eV; nm) and the oscillator strength. Comparison with experimental values is described later in the article (Section 3).

## 3. Experimental Analysis

**Figure 13:**
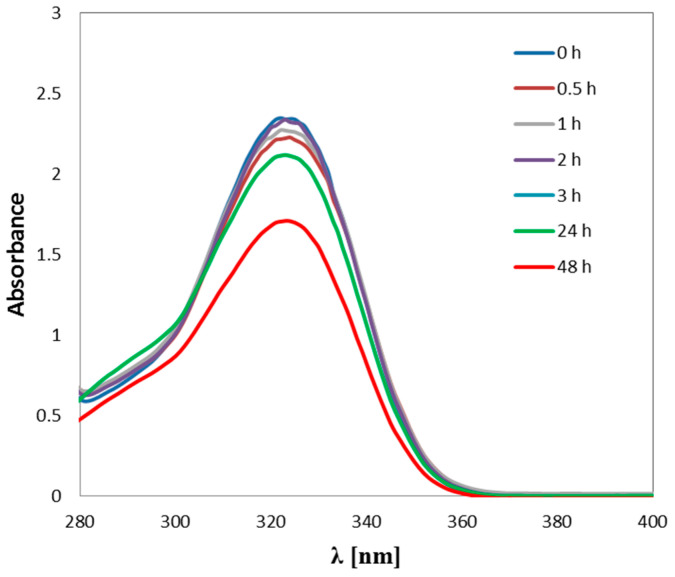
UV-Vis absorbance spectrum of mixture of carboplatin and pyridoxal phosphate (vit. B6) in an incubation buffer (1 mmol/L phosphate buffer, 4 mmol/L sodium-chloride, pH 7.4) [50].

UV-Vis spectroscopic techniques were used for physico-chemical characterization of interactions of B6 vitamin (pyridoxine hydrochloride) and carboplatin (Figure 13). The study was performed in the wavelength range from 190 nm to 500 nm.

Carboplatin and vitamin B6 with molar concentrations equal to 7.3 × 10−4 mol/L and 14.6 × 10−3 mol/L were prepared in the following incubation buffer: 1 mmol/L phosphate buffer and 4 mmol/L sodium chloride with pH equal to 7.4. Maximum absorbance of vitamin B6 was obtained at 323 nm wavelength and was almost equal to 3.0, which corresponds to 0.721 mmol/L concentration of vitamin B6 in the incubation buffer (Figure 13). Vitamin B6 was incubated with carboplatin in a 2:1 ratio at 37 °C. The absorbance of the mixture was measured at appropriate time intervals of incubation, namely, 0 h, 0.5 h, 1 h, 2 h, 4 h, 24 h and 48 h. The addition of the solution of carboplatin to the solution of vitamin B6 in the buffer causes a decrease of maximum absorbance for the studied vitamin by about 20% relative to the value of absorbance equal to 2.3 (time 0 h). It reflects a decrease in vitamin B6 concentration to a value of 0.624 mmol/L. Such a decrease of absorbance of the studied vitamin is caused by the formation of a drug–vitamin complex. However, a significant decrease by about 30% in maximum absorbance from the baseline of the complex (time 0 h) was observed after 48 h (1.75 absorbance) and a decrease by about 40% in the maximum absorbance of vitamin B6.

In the same way and under the same conditions, the absorbance maxima of all tested B vitamins and guanine with oxaliplatin (synonyms: [SP-4-2-(1R-trans)]-(1,2-Cyclohexanediamine-N,N′)[ethanedioata(2-)-O,O]platinum; oxaliplatinum; (SP-4-2)-[(1R,2R)-Cyclohexane-1,2-diamine-κN,κN′]-[ethanedioato(2-)-κO1,κO2]platinum) were determined. The initial concentrations of substrates in the reaction of complexation with oxaliplatin were 0.503 mmol/L for guanine, 0.178 mmol/L for vitamin B1 (thiamine), 0.107 mmol/L for vitamin B2 (riboflavin), 0.712 mmol/L for vitamin B3 (niacin) and 0.365 mmol/L for vitamin B6 (pyridoxal phosphate). The starting concentration for oxaliplatin was 0.252 mmol/L.

**Figure 14:**
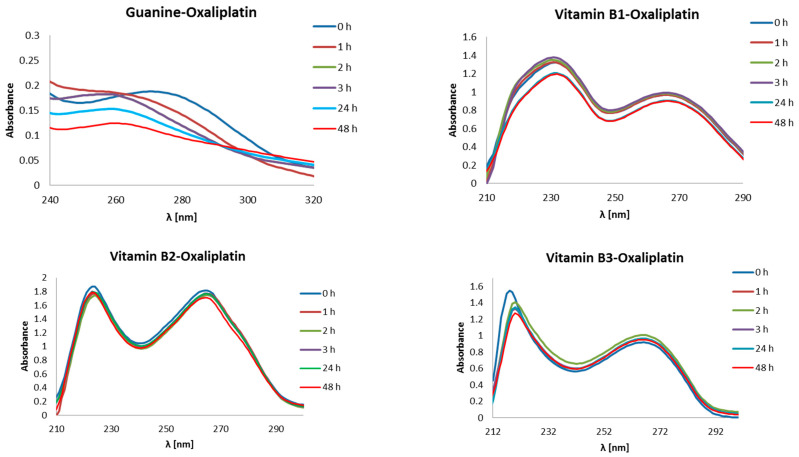

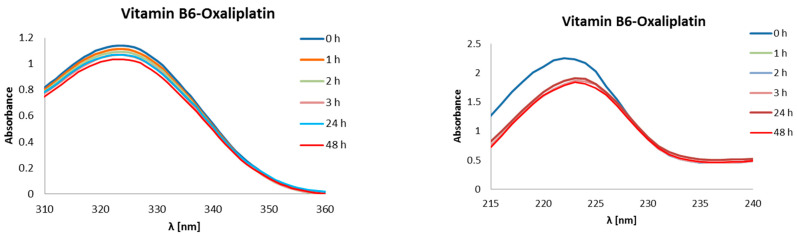
UV-Vis absorbance spectrum of mixture of oxaliplatin with B vitamins and guanine in an incubation buffer (1 mmol/L phosphate buffer, 4 mmol/L sodium-chloride, pH 7.4) [52].

Both guanine and all tested B vitamins in complexes with oxaliplatin show more than one maximum absorbance (Figure 14). Guanine has two maxima, at 218 nm and 271 nm. However, in the first case there are no changes observed in the height of the absorbance maximum after the formation of the base–drug complex in the subsequent time intervals tested. Guanine–oxaliplatin complex shows a 0.188 high absorbance maximum at 271 nm (0 h), which went down to 0.153 and 0.124 after 24 h and 48 h, respectively. However, with the passage of time, the absorbance maximum is shifted towards a shorter wavelength, namely, 242 nm. Similarly, as the nucleobase, vitamin B1 has two absorbance maxima of other wavelengths at 231 nm and 266 nm. Here, a decrease of absorbance maximum is observed from 1.314 (0 h) by 1.200 (24 h) to 1.119 (48 h) in the first case and from 0.967 (0 h) by 0.903 (24 h) to 0.897 (48 h) in the second case (Figure 14). Vitamin B2 has maxima at 224 nm and 265 nm, which decrease accordingly from 1.875 to 1.780 and from 1.818 to 1.710 after 48h, respectively. After 24 h, the maximum absorbance of complex vitamin B2–oxaliplatin gained heights of 1.790 at 224 nm and 1.770 at 265 nm. Vitamin B3 has a maximum at 218 nm and 266 nm at baseline. In first place, the absorbances were reduced from 1.548 to 1.344 after 24 h and to 1.277 after 48 h. At 266 nm no decrease was observed of the absorbance equal to 0.918. Vitamin B6 shows also two absorbance maxima at 222 nm and 323 nm (Figure 14). In the first case, a decrease was observed of maximum absorbance from 2.254 (0 h) by 1.857 (24 h) to 1.784 (48 h), while in the second case, an absorbance drop from 1.142 (0 h) by 1.070 (24 h) to 1.035 (48 h) was observed.

**Figure 15:**
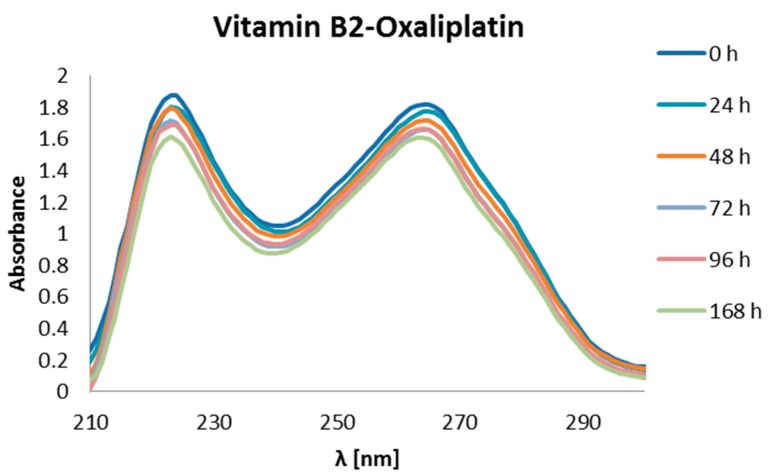
UV-Vis absorbance spectrum of mixture of oxaliplatin and riboflavin (vit. B2) in an incubation buffer (1 mmol/L phosphate buffer, 4 mmol/L sodium-chloride, pH 7.4) [52].

After 168 h of incubation in the buffer, the vitamin–oxaliplatin and base–oxaliplatin complexes did not decrease in their concentration in relation to the concentrations of the tested complexes after 48 h of incubation. Thus, after 7 days of incubation, the height of the absorbance peaks was comparable to the height after 48 h. The exception is vitamin B2, where there was a decrease in the maximum height by about 10% compared to the value tested after 48 h (Figure 15).

Experimental results reflect the results of ab initio calculations at the B3LYP/6-31G(d,p)/LANL2DZ level of calculation (GUA → vit. B3 → vit. B1 → vit. B6 → vit. B2).

**Figure 16:**
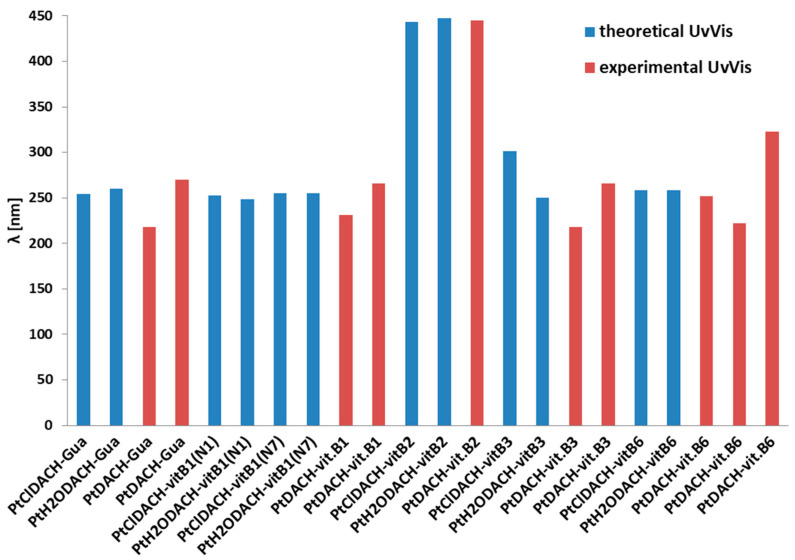
The experimental and the theoretical values of excitation energies (nm) of monoaqua and diaqua derivatives of oxaliplatin with B vitamin and guanine complexes [52].

In Figure 16 the experimental and the theoretical values of excitation energies are compared.

In the case of the complex with Guanine, the computed values are 254.22 nm and 260.50 nm for PtClDACH-Gua and for PtH_2_ODACH-Gua, respectively, while the experimental spectra showed close values of the absorbance maximum at 270 nm and at 218 nm (Figure 16). The PtClDACH-vit.B1(N_1_) and PtClDACH-vit.B1(N_7_) complexes have the maximum absorbance located at 252.64 nm and 255.04 nm, while PtH_2_ODACH-vit.B1(N_1_) and PtH_2_ODACH-vit.B1(N_7_) have it at 248.69 nm and 255.04 nm, respectively. The experimental data showed maxima of absorbance at 231 nm and 266 nm. The complexes with vitamin B2 showed this at 443.05 nm for PtClDACH-vit.B2 and 447.71 nm for PtH_2_ODACH-vit.B2, which is in a good agreement with experimental data locating the maximum absorbance bands at 445 nm (as well as at 221 nm, 266 nm and 372 nm). The calculated results of vitamin B3 showed the most intensive singlet-singlet transitions at 250.47 nm for PtH_2_ODACH-vit.B3 and at 301.58 nm for PtClDACH-vit.B3, while in the experimental spectrum the absorption maximum was found quite close at 266 nm and further at 218 nm. It was found that the absorbance maxima of computed absorption spectra for vitamin B6 occurred at 258.68 nm and at 258.55 nm for PtClDACH-vit.B6 and for PtH_2_ODACH-vit.B6, respectively. The experimental spectra showed a close absorbance maximum at 252 nm; however, maxima at 222 nm and 323 nm were found. The computed UV-Vis spectra are shown in Figure 12, while the theoretical and experimental excitation energy for the complex of drugs and the studied structures are shown in Figure 16. Very good agreement has been proven between computed and experimental UV-Vis spectra. Theoretical spectra were calculated in water solution, at the MN15/def2-TZVP level of theory with the application of the polarizable continuum model (PCM).

## 4. Conclusions

Theoretical and experimental studies were performed in order to describe the interactions of selected Pt (II) derivative drugs with nucleobases and other compounds of analogous structure, such as vitamins from B group: thiamine (vitamin B1), riboflavin (vitamin B2), niacin (vitamin B3) and pyridoxal phosphate (vitamin B6). cisplatin, oxaliplatin and carboplatin were tested. Ab initio studies on complexes of nucleobases/B vitamins with drugs such as cisplatin, oxaliplatin and carboplatin were based on DFT and TD-DFT methods. Theoretical research was supported by UV-Vis spectrophotometric experimental studies. The performed research on the affinity of nucleobases and their competitive B vitamins to drugs explains not only their mechanism of action at the molecular level, but also explains their competitiveness and impact on anticancer therapy. During the study, the reactivity of hydrolysis products of studied drugs were analyzed such as mono- and diaqua derivatives, (cis~[Pt(NH_3_)_2_Cl(H_2_O)]^+^) and (cis~[Pt(NH_3_)_2_(H_2_O)_2_]^2+^) in the case of cisplatin, [PtH_2_OClDACH]^+^ and [Pt(H_2_O)_2_DACH]^2+^ in the case of oxaliplatin and Pt(NH_3_)_2_H_2_O(OH)^+^ plus Pt(NH_3_)_2_(OH)_2_ in the case of carboplatin, which can interact not only with nucleobases, which is the basis of their therapeutic action, but also with all compounds which have aromatic rings in their structures. The research was carried out on two levels of calculation, namely, at the B3LYP/6-31G(d,p) and at the MN15/def2-TZVP levels of theory and their results were also compared with each other. The order of affinity of cisplatin and oxaliplatin for both monoaqua and diaqua derivatives of nucleobases and B vitamins is GUA → vit. B3 → vit. B6 → vit. B1_(N1)_ → vit. B1_(N7)_ → vit. B2, obtained at the B3LYP/6-31G(d,p) level of theory. However, in the case of cis~monoaqua derivatives, vitamin B6 alternates with vitamin B1 as GUA → vit. B3 → vit. B1_(N1)_ → vit. B1_(N7)_ → vit. B6 → vit. B2. With the changing of the calculation level to a higher one, the MN15/def2-TZVP level of theory, the affinity order changes to vit. B1_(N1,N7)_ → GUA → vit. B6→ vit. B2 → vit. B3, as seen in the example of oxaliplatin. Carboplatin shows weak affinity to the studied structures, except for guaniane and vitamin B6, in both mono- and diaqua derivatives. Vitamin B3 weakly interacts with the products of carboplatin hydrolysis. Other vitamins do not interact with this drug. Theoretical studies confirm clinical observations and indicate high therapeutic effectiveness of oxaliplatin and then cisplatin in anticancer treatment, as well as confirm the competitiveness of B vitamins in relation to nucleobases. The presence of intramolecular non-covalent interactions stabilizing the conformation of the drug–nucleobase/B vitamin complexes was confirmed via the application of the AIM theory. In addition, on the basis of the calculated and experimental spectra, the agreement between theoretical and experimental studies was proved. A decrease of concentration in the solutions of nucleobases or vitamins from B group was observed after their complexation with the tested Pt (II) drugs. However, it should be emphasized with certainty that B vitamins form weaker complexes with the products of hydrolysis of chemotherapeutics in relation to nucleobases.

## 5. Future Directions

The research on the stability of Pt (II) derivatives–B vitamins complexes and their physicochemical, thermodynamic and spectroscopic properties will be the main source of knowledge for a better understanding of the reactivity of these drugs with physiological target molecules such as nucleobases. This will open up further research on the behavior of other Pt (II) derivatives in this respect and will open up further studies for new potential Pt (II) drugs and drug delivery agents. In effect, all involved surveys will lead to the final results, that is, obtaining new alternative Pt (II) drugs and introducing new potential therapies used in patients with various types of cancer by using nanostructures as nanocarriers during targeted drug delivery, and will be completed with the synthesis of new potential medicines. The described results of potential interactions of Pt (II) anticancer drugs with compounds other than the ones in the target sites will contribute to the dissemination of knowledge about the use of an appropriate diet during chemotherapy.

## Figures and Tables

**Table 1:** The energetic characteristics of cis-Pt-Chloroaqua, Pt-DACH-Chloroaqua and Pt(NH_3_)_2_H_2_O(OH)^+^ in the case of cisplatin, oxaliplatin and carboplatin, respectively reactions with vitamins from B group and nucleobase as guanine obtained at the B3LYP/6-31G(d,p)/LANL2DZ level of theory in water solution. All energies are given in kcal/mol. Symbol Pt* stands for Pt(NH_3_)_2_Cl of cisplatin, Pt** stands for PtClDACH of oxaliplatin and Pt*** stands for Pt(NH_3_)_2_(H_2_O) of carboplatin. Red bold text refers to reactions with oxaliplatin and blue to reactions with carboplatin [49,50,51,52].

ΔG_r_ (kcal/mol)			Reaction
Cisplatin	Oxaliplatin	Carboplatin	
−18.90	−13.87	149.53	cis[Pt*/**/***]^+/+/2+^ + [B1_(N7)_]^+^ → cis[Pt/**/***-B1_(N7)_]^2+/2+/3+^
−20.89	−16.24	144.60	cis[Pt*/**/***]^+/+/2+^ + [B1_(N1)_]^+^ → cis[Pt/**/***-B1_(N1)_]^2+/2+/3+^
−3.75	−1.46	233.04	cis[Pt*/**/***]^+/+/2+^ + B2_(N7)_ → cis[Pt/**/***-B2_(N7)_]^+/+/2+^
−24.90	−22.32	−6.3	cis[Pt*/**/***]^+/+/2+^ + B3_(N7)_ → cis[Pt/**/***-B3_(N7)_]^+/+/2+^
−15.61	−17.06	−12.96	cis[Pt*/**/***]^+/+/2+^ + B6_(N7_) → cis[Pt/**/***-B6_(N7)_]^+/+/2+^
−28.17	−33.46	−30.89	cis[Pt*/**/***]^+/+/2+^ + GUA_(N7)_→cis[Pt/**/***-GUA_(N7_)]^+/+/2+^

**Table 2:** The energetic characteristics of the Pt-DACH-Chloroaqua reactions with vitamin B and nucleobases obtained at the MN15/def2-TZVP level of theory. All energies are given in kcal/mol. Symbol Pt* stands for PtClDACH [52].

Number of Reaction	ΔG_r_	Reaction
1	−46.48	[Pt*]^+^ + [B1_(N7)_]^+^ → [Pt*-B1_(N7)_]^2+^
2	−48.91	[Pt*]^+^ + [B1_(N1)_]^+^ → [Pt*-B1_(N1)_]^2+^
3	−41.55	[Pt*]^+^ + B2 → [Pt*-B2]^+^
4	−39.37	[Pt*]^+^ + B3 → [Pt*-B3]^+^
5	−45.93	[Pt*]^+^ + B6 → [Pt*-B6]^+^
6	−46.10	[Pt*]^+^ + GUA → [Pt*-GUA]^+^

**Table 3:** The energetic characteristics of the cis-Pt-diaqua in case of Cisplatin, the Pt-DACH-diaqua in case of Oxaliplatin and [Pt(NH_3_)_2_(OH)]^+^ reactions with vitamins B and nucleobases obtained at the B3LYP/6-31G(d,p)/LANL2DZ level of theory in water solution. All energies are given in kcal/mol. Symbol Pt* stands for Pt(NH_3_)_2_(H_2_O) of Cisplatin, Pt** for PtH_2_ODACH of Oxaliplatin and Pt*** for Pt(NH_3_)_2_(OH) of Carboplatin. Red bold text refers to reactions with products of Oxaliplatin, blue bold text refers to reactions with products of Carboplatin [49,52].

ΔG_r_ (kcal/mol)			Reaction
Cisplatin	Oxaliplatin	Carboplatin	
−20.23	−25.10	149.53	cis[Pt*/**/***]^2+/2+/+^ + [B1_(N7)_]^+^ → cis[Pt/**-B1_(N7)_]^3+/3+/2+^
−22.64	−30.15	144.60	cis[Pt*/**/***]^2+/2+/+^ + [B1_(N1)_]^+^ → cis[Pt/**-B1_(N1)_]^3+/3+/2+^
−7.84	−13.64	233.04	cis[Pt*/**/***]^2+/2+/+^ + B2_(N7)_ → cis[Pt/**-B2_(N7)_]^2+/2+/+^
−27.28	−35.53	−6.3	cis[Pt*/**/***]^2+/2+/+^ + B3_(N7)_ → cis[Pt/**-B3_(N7)_]^2+/2+/+^
−24.66	−30.60	−12.96	cis[Pt*/**/***]^2+/2+/+^ + B6_(N7_) → cis[Pt/**-B6_(N7)_]^2+/2+/+^
−37.58	−50.03	−30.89	cis[Pt*/**/***]^2+/2+/+^ + GUA_(N7)_→cis[Pt/**-GUA_(N7_)]^2+/2+/+^

**Table 4:** The energetic characteristics of the Pt-DACH-diaqua reactions with vitamin B and nucleobases obtained at the MN15/def2-TZVP level of theory. All energies are given in kcal/mol. Pt** symbol is PtH_2_ODACH [52].

Number of Reaction	ΔG_r_	Reaction
1	−46.45	[Pt**]^2+^ + [B1_(N7)_]^+^ → [Pt**-B1_(N7)_]^3+^
2	−50.58	[Pt**]^2+^ + [B1_(N1)_]^+^ → [Pt**-B1_(N1)_]^3+^
3	−42.96	[Pt**]^2+^ + B2 → [Pt**-B2]^2+^
4	−40.61	[Pt**]^2+^ + B3 → [Pt**-B3]^2+^
5	−47.11	[Pt**]^2+^ + B6 → [Pt**-B6]^2+^
6	−51.01	[Pt**]^2+^ + GUA → [Pt**-GUA]^2+^

**Table 5:** BCPs (bond critical points) and potential energy density calculated at the MN15/def2-TZVP level of theory [52].

BCP	PtClDACH-Gua			BCP	PtH_2_ODACH-Gua		
	ρ_BCP_(e×a_0_^−3^)	∇^2^ρ_BCP_(e×a_0_^−5^)	V (r)		ρ_BCP_(e×a_0_^−3^)	∇^2^ρ_BCP_(e×a_0_^−5^)	V (r)
Cl-Pt	1.02 × 10^−1^	1.97 × 10^−1^	-1.27 × 10^−1^	H_2_O-Pt	9.54 × 10^−2^	4.69 × 10^−1^	−1.65 × 10^−1^
N-Pt	1.25 × 10^−1^	4.53 × 10^−1^	−2.06 × 10^−1^	N-Pt	1.28 × 10^−1^	4.31 × 10^−1^	−2.07 × 10^−1^
H_2_N-Pt	1.25 × 10^−1^	4.12 × 10^−1^	−1.96 × 10^−1^	H_2_N-Pt	1.39 × 10^−1^	3.77 × 10^−1^	−2.12 × 10^−1^
H_2_N-Pt	1.26 × 10^−1^	4.15 × 10^−1^	-1.99 × 10^−1^	H_2_N-Pt	1.27 × 10^−1^	4.04 × 10^−1^	−1.99 × 10^−1^
O…HN	1.84 × 10^−2^	7.27 × 10^−2^	−1.23 × 10^−2^	O…HN	1.90 × 10^−2^	7.36 × 10^−2^	−1.27 × 10^−2^
